# Three-Weekly Doses of Azithromycin for Indigenous Infants Hospitalized with Bronchiolitis: A Multicentre, Randomized, Placebo-Controlled Trial

**DOI:** 10.3389/fped.2015.00032

**Published:** 2015-04-21

**Authors:** Gabrielle B. McCallum, Peter S. Morris, Keith Grimwood, Carolyn Maclennan, Andrew V. White, Mark D. Chatfield, Theo P. Sloots, Ian M. Mackay, Heidi Smith-Vaughan, Clare C. McKay, Lesley A. Versteegh, Nerida Jacobsen, Charmaine Mobberley, Catherine A. Byrnes, Anne B. Chang

**Affiliations:** ^1^Child Health Division, Menzies School of Health Research, Charles Darwin University, Darwin, NT, Australia; ^2^Department of Paediatrics, Royal Darwin Hospital, Darwin, NT, Australia; ^3^Menzies Health Institute Queensland, Griffith University and Gold Coast University Hospital, Gold Coast, QLD, Australia; ^4^Department of Paediatrics, Townsville Hospital, Townsville, QLD, Australia; ^5^Queensland Paediatric Infectious Diseases Laboratory, Queensland Children’s Medical Research Institute, Sir Albert Sakzewski Virus Research Centre, Children’s Health Queensland Hospital and Health Service, University of Queensland, Herston, QLD, Australia; ^6^Clinical Medical Virology Centre, School of Chemistry and Molecular Biosciences, University of Queensland, St Lucia, QLD, Australia; ^7^The University of Auckland and Starship Children’s Hospital, Auckland, New Zealand; ^8^Queensland Children’s Medical Research Institute, Children’s Health Queensland, Queensland University of Technology, Brisbane, QLD, Australia

**Keywords:** bronchiolitis, Indigenous, viruses, bacteria, respiratory syncytial virus, macrolides, azithromycin, randomized controlled trial

## Abstract

**Background:**

Bronchiolitis is a major health burden in infants globally, particularly among Indigenous populations. It is unknown if 3 weeks of azithromycin improve clinical outcomes beyond the hospitalization period. In an international, double-blind randomized controlled trial, we determined if 3 weeks of azithromycin improved clinical outcomes in Indigenous infants hospitalized with bronchiolitis.

**Methods:**

Infants aged ≤24 months were enrolled from three centers and randomized to receive three once-weekly doses of either azithromycin (30 mg/kg) or placebo. Nasopharyngeal swabs were collected at baseline and 48 h later. Primary endpoints were hospital length of stay (LOS) and duration of oxygen supplementation monitored every 12 h until judged ready for discharge. Secondary outcomes were: day-21 symptom/signs, respiratory rehospitalizations within 6 months post-discharge and impact upon nasopharyngeal bacteria and virus shedding at 48 h.

**Results:**

Two hundred nineteen infants were randomized (*n* = 106 azithromycin, *n* = 113 placebo). No significant between-group differences were found for LOS (median 54 h for each group, difference = 0 h, 95% CI: −6, 8; *p* = 0.8), time receiving oxygen (azithromycin = 40 h, placebo = 35 h, group difference = 5 h, 95% CI: −8, 11; *p* = 0.7), day-21 symptom/signs, or rehospitalization within 6 months (azithromycin *n* = 31, placebo *n* = 25 infants, *p* = 0.2). Azithromycin reduced nasopharyngeal bacterial carriage (between-group difference 0.4 bacteria/child, 95% CI: 0.2, 0.6; *p* < 0.001), but had no significant effect upon virus detection rates.

**Conclusion:**

Despite reducing nasopharyngeal bacterial carriage, three large once-weekly doses of azithromycin did not confer any benefit over placebo during the bronchiolitis illness or 6 months post hospitalization. Azithromycin should not be used routinely to treat infants hospitalized with bronchiolitis.

**Clinical trial registration:**

The trial was registered with the Australian and New Zealand Clinical Trials Register: Clinical trials number: ACTRN1261000036099.

## Introduction

Bronchiolitis is the most common acute viral lower respiratory infection in infants worldwide causing more than three million hospitalizations annually (1). Indigenous children from affluent countries, such as Australia and New Zealand, are at particular risk. They are more likely than non-Indigenous children to be hospitalized (2, 3), to have longer hospital stays (2), to receive antibiotics for pneumonia (2, 4), and to be rehospitalized in the next 6 months with a respiratory illness (5). Indigenous children also have high rates of pneumonia and bronchiectasis (the latter related to recurrent pneumonia) (6, 7) and their upper airways are colonized with bacterial pathogens from an early age (8).

Supportive care, including supplemental oxygen, respiratory support, and fluid replacement, underpins bronchiolitis management. Clinical trials have shown bronchodilators, mucolytics, anti-viral, and anti-inflammatory agents to be ineffective (9). However, macrolide antibiotics pose as an attractive alternative, especially for Indigenous infants with their high risk of severe disease and secondary bacterial complications (5). In addition to possessing direct antibacterial actions, including activity against *Mycoplasma pneumoniae* and *Chlamydiales* species, macrolides modulate macrophage, neutrophil, and epithelial cell function *in vitro* and in experimental models (10), and possess potential anti-viral properties. Clarithromycin reduces respiratory syncytial virus (RSV) receptor numbers on epithelial cell surfaces; while azithromycin induces interferon-stimulated genes in rhinovirus (RV)-infected bronchial epithelial cells (11, 12). Finally, a single azithromycin dose decreases nasopharyngeal bacterial loads (5) and transiently reduces the risk of acute lower respiratory infections in African children following mass trachoma prevention campaigns or when contributing to combination therapy for malaria (13, 14).

Four placebo, double-blind, randomized controlled trials (RCTs) have evaluated the efficacy of macrolides in children hospitalized with bronchiolitis (5, 15–17). The first trial from Turkey (15), involving 21 infants aged ≤7 months with RSV, found that clarithromycin daily for 3 weeks significantly reduced hospital length of stay (LOS) and supplemental oxygen and intravenous fluid requirements compared to placebo. In contrast, 3 later RCTs (5, 16, 17) involving a total of 352 infants (up to age 2 years) with clinically diagnosed bronchiolitis from Australia, the Netherlands, and Brazil failed to demonstrate any clinical benefit using azithromycin for 1, 3, or 7 days, respectively. The Turkish trial was also the only one where treatment extended beyond the period in hospital where just 1 of the 12 infants in the clarithromycin group was re-hospitalized compared with 4 of 9 receiving placebo (15).

At the time we started our study, only the RCTs from Turkey (15) and the Netherlands (16) had been completed. We believed Indigenous infants were more like high-risk children in Turkey and might also benefit from a longer treatment course (the Turkish study was the only one to have addressed this question) (15). This is of considerable importance for Indigenous infants where respiratory symptoms and recurrent hospitalized respiratory illnesses are major risk factors for developing bronchiectasis (5–7). As administering twice-daily clarithromycin on an ambulatory basis is impractical in our setting (18), we took advantage of azithromycin’s long half-life and favorable pharmacokinetics by opting to determine whether a longer course (three large once-weekly doses) of azithromycin for Indigenous infants hospitalized with bronchiolitis improved clinical outcomes (LOS and duration of oxygen supplementation). Secondary aims were to: (i) determine the effects of azithromycin on respiratory symptoms and signs at day-21 and respiratory hospitalizations in the following 6-month period; (ii) assess the short-term impact of azithromycin upon nasopharyngeal carriage and respiratory virus shedding and; (iii) describe the viruses, *M. pneumoniae* and *Chlamydiales* species detected in these infants.

## Materials and Methods

### Study design and setting

This was a multi-center, randomized, double-blind, placebo-controlled trial. Infants were recruited at the Royal Darwin (June 2010–September 2013) and Townsville Hospitals (August 2010–June 2013), Australia and the Auckland Starship Children’s Hospital (November 2012–September 2013), New Zealand. Human Research Ethics Committees at all participating institutions approved the study and caregivers provided written, informed consent. The study was registered with the Australian and New Zealand Clinical Trials Register: ACTRN12608000150347 and monitored by an independent data safety monitoring board (DSMB).

### Participants

As described in detail previously (19), eligible infants were aged ≤24 months and hospitalized with a standardized clinical diagnosis of bronchiolitis (age-adjusted tachypnea with wheeze or crackles), had parent-ascribed Indigenous ethnicity (Australian Aboriginal, Torres Strait Islander, Maori, and/or Pacific Islander), were consented within 24 h of hospitalization and had caregivers with a mobile phone (see supplement for exclusion criteria and tachypnea definitions).

### Randomization, allocation and blinding, and medications

An independent statistician used a computer-generated, permuted block design to generate randomization sequences. Sealed opaque envelopes (selecting one of the eight different bottle codes) concealed the treatment allocation. Infants were allocated in a 1:1 ratio, stratified by age (≤6 or >6 months), oxygen supplementation on presentation (yes/no) and site (Darwin, Townsville, or Auckland), to once-weekly doses of oral azithromycin or placebo for 3 weeks. Neither the study team (researchers, hospital staff) nor parents were aware of assigned treatment groups until data analysis was completed.

The first dose was given in the hospital (30 mg/kg azithromycin or equal volume of placebo). The remaining two doses were supervised directly by study nurses (urban-based participants) or given at home by caregivers (remote-based participants) at weekly intervals. Study nurses contacted families via mobile phones to help ensure adherence (20).

### Clinical assessment, management, and outcomes

Demographic, medical history, and clinical data were recorded on standardized data collection forms. A validated severity score was employed (see Supplementary Material). Infants with bronchiolitis were managed at each site according to a common protocol, which outlined when supplementary oxygen was indicated (SpO_2_ <94%) and when nasogastric feeds or intravenous fluids were required. The protocol was in place for several months before commencing the trial. Infants received additional therapies (other than macrolides) at the discretion of the treating pediatrician.

The primary endpoints of LOS for respiratory illness and duration of oxygen requirement (where applicable) were monitored every 12 h. LOS was the time from admission to time “ready for discharge” from respiratory care as defined by SpO_2_ >94% in air for >16 h and feeding adequately. In our setting, “ready for discharge” from respiratory care can differ from LOS, as discharge from hospital may be delayed because of non-medical factors (such as waiting for air transport back to remote communities). For the other clinical outcomes, the day-21 review was conducted by study nurses (urban-based participants) and local health clinic staff (remote-based). Respiratory rehospitalization within 6 months of discharge was recorded through community and hospital electronic records. Adverse events were monitored daily in hospital by research staff and following discharge with weekly phone calls until the day-21 review.

### Specimen collection and processing

Nasopharyngeal swabs (NPS) taken before initial study medications were administered and repeated 48 h later, and were processed as described previously (21–23) for viruses and atypical bacteria (*C. pneumoniae*, *Simkania negevensis*, *M. pneumoniae*) using real-time polymerase chain reaction (PCR) assays (see Supplementary Material for list of viruses). NPS were cultured for respiratory bacterial pathogens that also underwent antibiotic susceptibility testing (24).

### Sample size and analysis

Based upon our previous data where the mean LOS in Indigenous infants with bronchiolitis was 96 (SD 24) hours (2), we estimated a total sample of 200 infants (100 in each age sub-group: ≤6 months and >6 months) would provide 94% power to detect a difference in the mean LOS of 12 h between treatment groups at the 5% significance level (two-tailed) and 95% power to detect a reduction in respiratory rehospitalization within the next 6 months from 30 to 10% (19).

Data were analyzed according to our published protocol. Data were analyzed according to the group the child was allocated to. Only available data were analyzed. Between-group differences were tested using Fisher’s exact test (for proportions) and Mann–Whitney *U* test (for continuous variables). A 95% confidence interval (CI) was obtained for the difference in medians between treatment groups (25). Subgroup analysis was performed by age (≤6 and >6 months) as planned (19), and also for groups based on oxygen requirement when enrolled, remoteness, antibiotic use, and previous respiratory hospitalizations for the three clinical outcomes. These *post hoc* subgroup analyses were conducted that might inform clinical practice. *p*-Values are reported for the subgroup × treatment interaction term in a linear regression model (for log-transformed LOS and duration of supplemental oxygen) and in a logistic regression model (for any readmissions within 6 months) with main effects for just treatment group and subgroup. Data were also analyzed adjusting for significant between-group differences at baseline.

## Results

**Figure 1:**
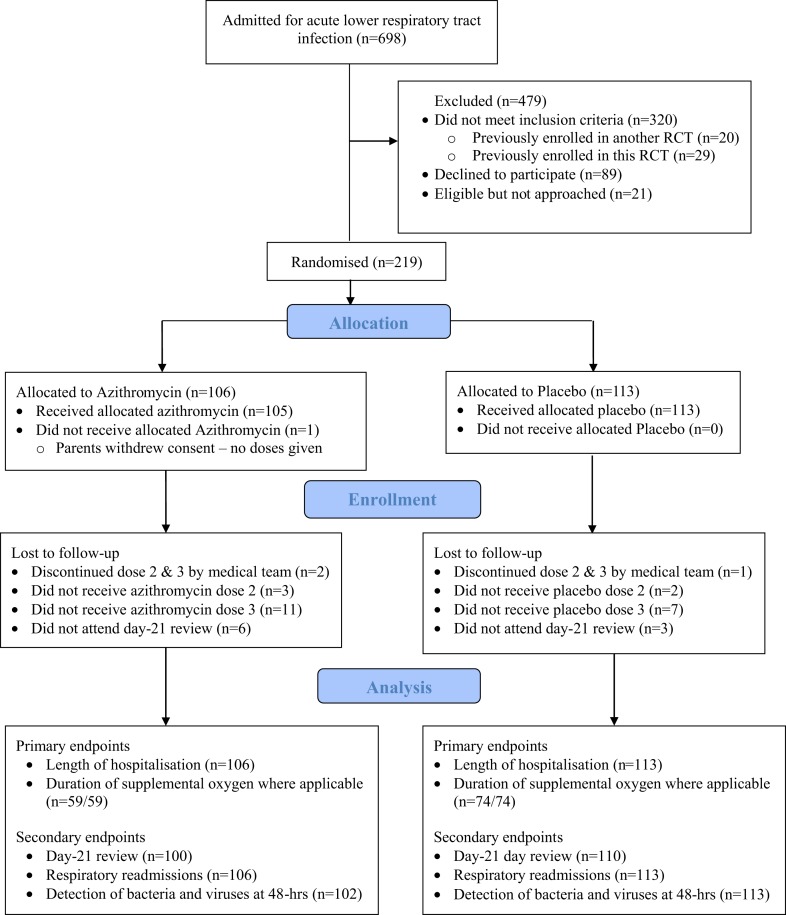
**CONSORT flow diagram**.

We recruited 219 infants (106 randomized to azithromycin, 113 to placebo (Figure 1). Overall, 218 received dose-1 in hospital; 102 (azithromycin) and 111 (placebo) received dose-2 and 94 (azithromycin); and 106 (placebo) received dose-3.

Demographical and clinical characteristics were similar between treatment groups, apart from household smoke exposure involving more infants in the azithromycin (69%) than the placebo (50%) group, *p* = 0.01; see Table 1). Of the study cohort, 133 (61%) required oxygen during hospitalization. Non-macrolide antibiotics were prescribed in 93 (43%) infants before hospitalization (see Supplementary Material) and none received steroids or required intensive care management. Thirty-eight infants were hospitalized previously for a respiratory illness (azithromycin: 18; placebo: 20). Retention was high (≥97%) for the clinical endpoint at day-21.

**Table 1:** **Demographic and clinical characteristics of 219 patients randomized to treatment with either azithromycin (*n* = 106) or placebo (*n* = 113)**.

	Azithromycin (*n* = 106)	Placebo (*n* = 113)
Age (months)	5.7 (3–10)	5.6 (3–9)
≤6 months	56 (53%)	61 (54%)
6–24 months	50 (48%)	52 (46%)
Male	64 (60%)	72 (64%)
Indigenous Australians	92 (87%)	95 (84%)
New Zealand Maori/Pacific Islander	14 (13%)	18 (16%)
Gestational age (weeks)	38 (36–40)	38 (36–40)
Birth weight (kg)	3.03 (2.3–3.3)	3.00 (2.5–3.4)
Remote region	74 (70%)	71 (63%)
Currently breastfed	82 (78%)	85 (75%)
Mother smoked during pregnancy	54 (51%)	58 (51%)
Exposed to household smoke	72 (69%)	57 (50%)
Previously hospitalized for acute respiratory infection	18 (17%)	20 (18%)
Symptoms leading up to admission (reported by parents)
Number of days with symptoms	3 (2–4)	3 (2–4)
Nasal discharge	93 (89%)	96 (85%)
Cough	104 (99%)	110 (97%)
Breathing difficulties	102 (97%)	111 (98%)
Poor feeding	48 (46%)	63 (56%)
Lethargy	52 (50%)	52 (46%)
Baseline clinical severity score (see Supplementary Material)	5 (4–7)	5 (3–7)
Enrollment observations
Number receiving oxygen	59 (56%)	74 (65%)
Level of supplemental oxygen (L/min)	1 (0.5, 2.0)	1 (0.5, 1.5)
Heart rate (bpm)	152 (137, 164)	147 (138, 156)
Temperature (°C)	36.6 (36.4, 36.9)	36.7 (36.4, 37.0)
Non-macrolide antibiotics prescribed prior to hospital	47 (45%)	46 (42%)
Non-macrolide antibiotics prescribed during hospital	64 (61%)	68 (60%)
Supplemental intravenous fluid administered	24 (23%)	23 (20%)
Chest radiograph taken	87 (83%)	88 (78%)
Co-morbidities
Any otitis media	21 (20%)	18 (16%)
Otitis media with perforation	4 (4%)	6 (5%)
Skin infection	32 (30%)	27 (24%)
Anemia	9 (9%)	4 (4%)
Failure to thrive	5 (5%)	6 (5%)
Lobar pneumonia/atelectasis on Chest X-ray	21 (20%)	13 (12%)
Any virus detected	82 (79%)	92 (83%)
Any respiratory bacterial pathogen detected	73 (70%)	83 (73%)

*Data are presented as median and inter-quartile range for continuous variables, or actual numbers (%) for categorical variables*.

*NB: co-morbidities as documented from hospital admitting medical team. Missing variables described below*.

*Azithromycin: gestational age = 7 (7%), birth weight = 11 (10%), mother smoked during pregnancy = 4 (4%), exposure to household smoke = 2 (2%), breathing difficulties = 2 (2%), poor feeding = 3 (3%), lethargy = 12 (11%), enrollment temperature = 2 (2%)*.

**Placebo*: gestational age = 7 (6%), birth weight = 9 (8%), number of days with symptoms = 1 (1%), nasal discharge = 4 (4%), breathing difficulties = 1 (1%), lethargy = 10 (9%), enrollment pulse = 1 (1%), temperature = 3 (3%), respiratory rate = 1 (1%)*.

### Clinical outcomes

**Figure 2:**
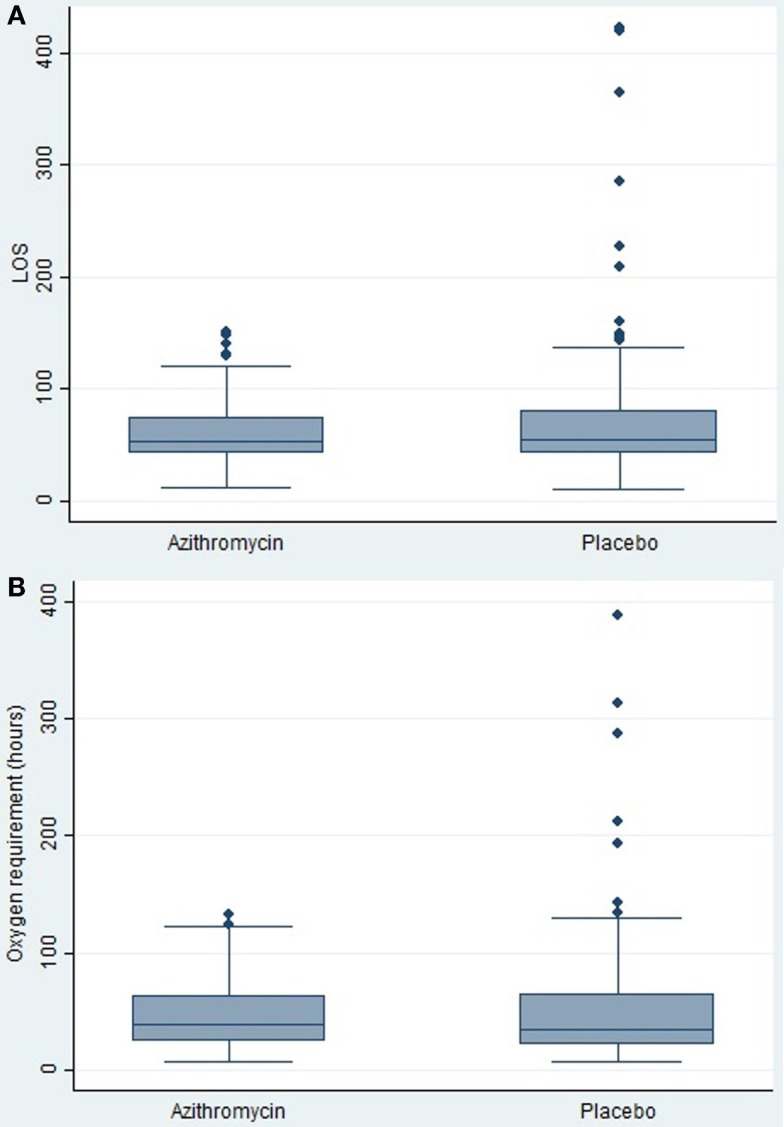
**(A)** Length of hospital stay (LOS) until ready for discharge from respiratory care. There was no significant difference between children randomized to azithromycin and placebo. **(B)** Time children received supplementary oxygen (where applicable). There was no significant difference between children randomized to azithromycin and placebo.

No significant between-group differences were found for LOS or duration of supplemental oxygen (Figures 2A,B). The median LOS of 54 h was identical in both groups (difference = 0 h, 95% CI: −6, 8; *p* = 0.8), while the median time receiving oxygen was 40 h in the azithromycin group and 35 h in the placebo group (difference = 5 h, 95% CI: −8, 11; *p* = 0.7).

### Day-21 clinical review and 6-month readmission

Two hundred ten (97%) infants completed the day-21 review (Figure 1). Although persistent symptoms or signs were more common in placebo group, the between-group differences were not significant (Table 2).

**Table 2:** **Persistent respiratory symptoms/signs at day-21 review**.

	Azithromycin *N* = 100 (%)	Placebo *N* = 110 (%)	Risk difference (95% CI)	*p*- value*
Wet cough	12 (12%)	17 (15%)	−4% (−12%, 5%)	0.4
Crackles/crepitations	7 (7%)	15 (14%)	−6% (−14%, 1%)	0.1
Chest recession	0 (0%)	2 (2%)	−2% (−4%, 0.7%)	0.2
Wheeze	11 (11%)	11 (10%)	1% (−7%, 9%)	0.9
Any of the above	23 (23%)	35 (32%)	−8% (−20%, 3%)	0.2

*Fisher’s exact test.

Overall, 81 (azithromycin *n* = 47, placebo *n* = 34) respiratory rehospitalizations were recorded from 56 participants (azithromycin *n* = 31, placebo *n* = 25). No significant between-group differences were found (odds ratio for any hospitalization 1.5, 95% CI: 0.8, 3.0, *p* = 0.2). Sixty (74%) rehospitalizations were for wheezing-associated illness.

There was no evidence of a differential effect of azithromycin on any of the main three clinical outcomes for any of the subgroups (see Tables S2–S4 in Supplementary Material). Adjustment for household smoking exposure had negligible effect on the trial’s main analyses (see Table S5 in Supplementary Material).

### Microbiology

Nasopharyngeal swabs were collected from 217 infants at baseline and 215 at 48 h. At baseline, at least 1 virus was detected in 174 (81%) infants (see Figure S1 in Supplementary Material). RSV was detected in 91 (42%), followed by HRV (79; 37%) and adenovirus (14; 7%). The mean number of viruses detected per infant hardly changed from baseline to 48 h; azithromycin: 1.0–0.9 viruses (difference 0.1, 95%CI: −0.2, 0.2; *p* = 0.4); placebo: 1.1–1.0 viruses (difference 0.1, 95%CI: −0.02, 0.3; *p* = 0.09).

Nasopharyngeal swabs isolations of *S. pneumoniae, non-typable H. influenzae*, and *M. catarrhalis* at 48 h were less common in the azithromycin group than in the placebo group (Table 3). On average, there were 0.4 (95% CI: 0.2, 0.6; *p* < 0.001) fewer bacterial species per infant in the azithromycin group.

**Table 3:** **Nasal swab bacteriology pre- and 48 h post-treatment**.

	Pre-treatment (baseline)		Post-treatment (48 h)			
	Azithromycin *n* = 104 (%)	Placebo *n* = 113 (%)	Azithromycin *n* = 102 (%)	Placebo *n* = 113 (%)	OR Azithromycin vs. placebo (95% CI)^a^	*p*-value**
*S. pneumoniae*	24 (23)	32 (28)	6 (6)	24 (21)	0.2 (0.06, 0.5)	0.001
Penicillin intermediate resistant	11 (11)	11 (10)	3 (3)	8 (7)	0.2 (0.05, 1.2)	0.2
Penicillin resistant	1 (1)	0 (0)	1 (1)	1 (1)	–	1.0
Azithromycin resistant	6 (6)	6 (5)	3 (3)	6 (5)	0.3 (0.03, 2.02)	0.5
*Non-typable H. influenzae*	38 (37)	43 (38)	10 (10)	34 (30)	0.2 (0.07, 0.4)	<0.001
Azithromycin resistant	0 (0)	1 (1)	0 (0)	0 (0)	–	–
Ampicillin resistant	8 (8)	8 (7)	1 (1)	9 (8)	0.02 (0.001, 0.4)	0.02
Beta-lactamase positive	8 (8)	8 (7)	1 (1)	9 (8)	0.02 (0.001, 0.4)	0.02
*M. catarrhalis*	33 (32)	50 (44)	12 (12)	41 (36)	0.2 (0.08, 0.5)	<0.001
Beta-lactamase positive	30 (29)	50 (44)	12 (12)	41 (36)	0.2 (0.1, 0.6)	0.001
*S. aureus*	11 (11)	15 (13)	10 (10)	17 (15)	0.6 (0.2, 1.6)	0.3
Erythromycin non-susceptible	2 (2)	6 (5)	4 (4)	7 (6)	0.8 (0.2, 3.0)	0.5
MRSA	2 (2)	4 (4)	1 (1)	6 (5)	0.1 (0.007, 2.0)	0.1

*OR, odds ratio*.

*Four infants not counted in table results (i.e., refused, missed)*.

*Minimum inhibitory concentrations determined for *S. pneumoniae* and non-typable *H. influenzae* (NTHi), and breakpoints defined using European Committee on Antimicrobial Susceptibility Testing (EUCAST) (http://www.eucast.org): *S. pneumoniae*, intermediate penicillin resistant, MIC >0.06–2 mg/L, penicillin resistant, MIC >2 mg/L, and azithromycin resistant MIC >0.5 mg/L; and for *H. influenzae*, azithromycin resistant, MIC >4 mg/L and ampicillin resistant, MIC >1 mg/L*.

*^a^Logistic regression – difference between treatment groups at post-treatment (48 h)*.

**p-value from Fisher’s exact test.

### Adverse events

Three adverse events were reported to our DSMB. In the azithromycin group, one infant presented to hospital with vomiting and diarrhea and another vomited the trial medication. In the placebo group, one infant presented to hospital with wheezing and a rash. All recovered and none discontinued the trial.

## Discussion

This is the first international, multicentre, double-blind RCT of an extended course of macrolides in bronchiolitis. In this study involving 219 Indigenous infants, we found that three once-weekly doses of azithromycin (compared to placebo), conferred no benefit in terms of LOS, duration of oxygen supplementation, day-21 symptom/signs, or respiratory rehospitalizations within 6 months post discharge. Azithromycin significantly reduced the mean number of nasopharyngeal bacteria per infant, but not the mean number of viruses per infant.

Our study is larger than the four preceding published studies on macrolides in bronchiolitis (5, 15–17) and involved a group of infants from populations at high risk for chronic suppurative lung disease (6). Our findings on the lack of beneficial effect of azithromycin for hospital-based outcomes (LOS and duration of oxygen supplementation) are concordant with three of these studies (5, 16, 17). To date, the Turkish study is unique (15) where 3 weeks of clarithromycin reported reduced LOS, oxygen supplementation, and respiratory rehospitalization within the following 6 months. Possible reasons for the difference between the Turkish (15) and other studies (5, 16, 17) include: using clarithromycin with its greater lung penetration and potential anti-RSV activity (11), differences in sample size, attrition population characteristics, the role of chance, and increased risk of bias associated with small studies.

We used a longer course of azithromycin than the other studies (5, 15–17) and did not find any clinically significant between-group outcomes. Azithromycin had no significant effect on the presence of persistent symptoms/signs on day-21 review and the proportion with persistent respiratory symptoms or signs (14–24%) are similar to another report of 25% infants remaining symptomatic after 21 days (26). Furthermore, the importance of the symptoms beyond hospitalization of persistent cough and wheeze was highlighted in a guideline on bronchiolitis (27).

The proportion of respiratory rehospitalizations within 6 months of discharge (25%) was also similar to other trials (5, 15). Rehospitalization for respiratory illness is an important outcome because it is an independent risk factor for bronchiectasis in Indigenous children (6). We targeted this high-risk group, as respiratory diseases are prevalent and more severe and persistent in this population (5, 28).

The mean number of nasopharyngeal respiratory bacteria was reduced more in the azithromycin than the placebo arm, as seen in our previous short-term RCT (5). Although the number of macrolide-resistant bacteria at 48 h also declined, this was not to the same extent as found in susceptible strains. Meanwhile, detection rates for respiratory viruses between treatment groups changed little over the 48 h following enrollment, though PCR detects nucleic acids, it is not possible to determine whether differences in viable viruses existed with azithromycin treatment.

Our study has several other limitations, which may have resulted in the negative results of our RCT. First, including an older age group increased the risk of including those with asthma. However, our cohort’s median age of 6 months (IQR 3, 9) reduced this possibility. Further, in both the UK and USA bronchiolitis guidelines, the upper age limit is 23–24 months. Second, in the Australian centers, we included those hospitalized previously for an acute respiratory infection, which may have contributed to the number of respiratory rehospitalizations (27, 29). Third, the concurrent use of antibiotics in two-thirds of infants with bronchiolitis may limit the ability of additional macrolide treatment to improve outcomes. We were unable to influence the clinical practice of physicians on this matter. However, subgroup analyses on previous respiratory hospitalization, or antibiotic use did not show any significant differences on any of our outcomes (see Supplementary Material). Fourth, our strategy of using three large once-weekly azithromycin doses may have produced sub-optimal results. This, however, is unlikely as prior RCTs of daily azithromycin found no benefit (16, 17). Moreover, in children, single large doses of azithromycin can successfully treat otitis media (18), while for several weeks after its mass distribution within rural African villages for controlling trachoma, azithromycin reduced the risk of acute lower respiratory infections by more than one-third (13). Although our RCT (18) used the same regime for long-term therapy of children with bronchiectasis and showed that azithromycin significantly reduced the exacerbation rate by 50% (compared to placebo), the different disease and younger age group in this RCT meant an alternative dose and/or regime for optimal efficacy may have been required. Finally, even though parents reported verbally that doses-2 and 3 were given, we were unable to directly observe these for remote-based infants (*n* = 145). However, the day-21 follow-up rate of >97% at the local health clinic implies that parents are likely to have adhered to the study protocol. This is supported by feedback from research nurses who interviewed parents throughout the trial.

Our study was aimed at infants at high risk of future bronchiectasis and employed a 3-week equivalent course in a multicentre setting. This design was to maximize the chances of demonstrating a clinical benefit for azithromycin in our target population, by reducing subsequent hospitalization and shortening hospital stay (both are independent risk factors for later development of bronchiectasis). Despite this, no advantage from receiving azithromycin was identified. Moreover, there are now growing concerns over the increasing global consumption of antibiotics and rising rates of antibiotic resistance, especially when few new anti-microbial agents are in the developmental pipeline (30, 31). Much of this antibiotic resistance is being driven by antibiotics prescribed for viral respiratory infections, most notably long-acting, broad-spectrum agents, including azithromycin (32, 33). An emerging fear for this young age group is that antibiotics may also adversely affect the developing intestinal “microbiome” with potential deleterious long-term effects upon gastrointestinal, immunological, and metabolic programing (34). Thus, given our study’s negative findings and the concerns over the association between azithromycin and increased carriage of macrolide-resistant pathogens (35), the increasing need for anti-microbial stewardship, potential immediate and long-term adverse events, and associated costs, it is clear that these factors outweigh any postulated, but still unproven benefits of macrolides in this patient population.

## Conclusion

In this RCT of Indigenous infants hospitalized with bronchiolitis, we found that three once-weekly doses of azithromycin significantly reduced nasopharyngeal bacterial carriage, but did not have any significant impact on viruses or short (reduced LOS or duration of oxygen supplementation) or long-term (decreased odds of persistent symptoms at day-21 or respiratory rehospitalization within 6 months of discharge) clinical benefits compared with placebo. In light of these results, similar findings in other studies, and fears over rising antibiotic resistance, azithromycin should not be used to treat infants hospitalized with viral bronchiolitis.

## Author Contributions

GM set up and managed the study, recruited participants, performed the data analysis, and drafted the manuscript. AC conceptualized the study. AC and KG co-drafted the manuscript. AC, PM, KG, TS, AW, IM co-designed the study and contributed to obtaining the grant, interpreted the data, and edited the manuscript. CB was responsible for overseeing all aspects of the trial in NZ. CM, AW, and CB were responsible for standardizing the management of bronchiolitis in their units, recruiting participants, and edited the manuscript. CM, LV, NJ, CM were responsible for recruiting participants and edited the manuscript. TS and IM assisted in the viral components of the study and edited the manuscript. HS-V assisted in the microbiological components of the study and edited the manuscript. MC assisted in the data analysis and edited the manuscript. All authors read and approved the final manuscript.

## Conflict of Interest Statement

The authors declare that they have no conflicts of interest relevant to this article to disclose. This study was funded by National Health and Medical Research Council (NHMRC) grants (605809) and supported by a Centre for Research Excellence in Lung Health of Aboriginal and Torres Strait Islander Children (1040830). Gabrielle B. McCallum is supported by a NHMRC scholarship (1055262); Anne B. Chang is funded by a NHMRC practitioner fellowship (545216 and 1058213). Heidi Smith-Vaughan is supported by a NHMRC Career Development Fellowship (1024175).

## Supplementary Material

The Supplementary Material for this article can be found online at http://journal.frontiersin.org/article/10.3389/fped.2015.00032

## Author Note

Protocol: a full study protocol can be accessed at www.trialsjournal.com “Randomized controlled trial of azithromycin to reduce the morbidity of bronchiolitis in Indigenous infants: a protocol.”

## Abbreviations

CI, confidence interval; DSMB, data safety monitoring board; HRV, human rhinovirus; LOS, length of stay; NHMRC, National Health and Medical Research Council; NPS, nasopharyngeal swab; PCR, polymerase chain reaction; RCT, randomized controlled trial; RSV, respiratory syncytial virus.
