# Supplementary material for: Three-Weekly Doses of Azithromycin for Indigenous Infants Hospitalized with Bronchiolitis: A Multicentre, Randomized, Placebo-Controlled Trial
Source: Front Pediatr. 2015 Apr 21;3:32. doi: 10.3389/fped.2015.00032 (PMC4404864; doi:10.3389/fped.2015.00032)

frontiers

Supplementary Material

Three-weekly doses of azithromycin for Indigenous infants hospitalised with bronchiolitis: A multicentre, randomised, placebo-controlled trial

Gabrielle B McCallum[1], Peter S Morris[1,2], Keith Grimwood[3], Carolyn Maclennan[2], Andrew V White[4], Mark D Chatfield[1], Theo P Sloots[5], Ian M Mackay[5,6], Heidi Smith-Vaughan[1], Clare C McKay[1], Lesley A Versteegh[1], N Jacobsen[4] , C Mobberley[7], Catherine A Byrnes[7], Anne B Chang[1,8]

[1]Child Health Division, Menzies School of Health Research, Charles Darwin University, Darwin, Northern Territory, Australia;
[2]Dept of Paediatrics, Royal Darwin Hospital, Darwin, Northern Territory, Australia;
[3] Menzies Heath Institute Queensland, Griffith University and Gold Coast University Hospital, Gold Coast, Queensland, Australia;
[4]Dept of Paediatrics, Townsville Hospital, Townsville, Queensland, Australia;
[5]Queensland Paediatric Infectious Diseases Laboratory, Queensland Children's Medical Research Institute, Sir Albert Sakzewski Virus Research Centre, Children's Health Queensland Hospital and Health Service, University of Queensland, Herston, Australia; [6]Clinical Medical Virology Centre, School of Chemistry and Molecular Biosciences, University of Queensland, St Lucia, Australia;
[7]The University of Auckland and Starship Children's Hospital, Auckland, New Zealand;
[8]Queensland Children's Respiratory Centre, Queensland Children's Medical Research Institute, Royal Children's Hospital, Brisbane, Queensland, Australia.

*** Correspondence:** Gabrielle B McCallum, Menzies School of Health Research, John Mathews Building, Bldg 58, Royal Darwin Hospital Campus, Rocklands Drive, Casuarina NT 0811, Australia, Phone: +61 8 89228588, Fax: +61 8 89227876, Email: gabrielle.mccallum@menzies.edu.au

1. Supplementary Data

METHODS

Exclusions were: severe disease (admitted to the intensive care unit); underlying chronic lung or congenital heart disease, contraindications to macrolides (e.g. hypersensitivity or liver dysfunction,), diarrhoea (>2 two watery stools above the normal daily pattern), received macrolides within last seven-days, or clinical and radiographic features of a primary pneumonia.[1] In New Zealand, infants with previous wheezing illnesses were excluded due to the high incidence of asthma in Maori and Pacific Islander children.[2]

Age-adjusted upper levels of normal respiratory rates were (i) respiratory rate $\geq$60/min if aged <2-months; (ii) $\geq$50/min if 2-12 months; and (iii) >40/min if 13-24 months.

Clinical assessment

A previously validated severity score was assigned to each infant, and consisted of four components (respiratory rate, accessory muscle use, degree of wheezing and pulse oximetery saturation reading.[3] Each component scored between 0 and 3, providing a composite score between 0–12 (Supplementary Table-1).

Specimen collection and processing

NPS were processed for a broad panel of viruses RSV A and B; adenovirus, parainfluenza (1-3), influenza A and B, rhinovirus, human metapneumovirus, human coronaviruses (OC43, HKU1, 229E, NL63), human bocavirus, *C. trachomatis, C. pneumoniae*, *Simkania negevensis* and *M. pneumoniae*).[4]

Medications

The placebo, manufactured by the Institute of Drug Technology (IDT) (Melbourne, Victoria, Australia), had a similar look, smell and taste to active azithromycin. Azithromycin (Pfizer, Australia) was purchased and repackaged by IDT. Both medications were prepared as a powder in identical opaque bottles and sealed with an aluminium foil.

RESULTS

Antibiotics given prior to hospital include intramuscular ceftriaxone (n=47, 44%) and procaine penicillin (n=21, 20%) and oral amoxicillin (n=12, 11%). Additional non-macrolide antibiotics were also prescribed commonly during hospitalisation (n=132, 61%); oral amoxicillin was the most common (n=75, 33%), followed by parenteral benzylpenicllin (n=65, 29%) and oral amoxicillin-clavulanate (n=26, 11%).

References

1. Chang AB, Torzillo PJ, Boyce NC, et al. Zinc and Vitamin-A supplementation in Indigenous children hospitalised with episodes of lower respiratory tract infection: a randomised controlled trial. *Med J Aust* (2006) **184**: 107-12.
2. Ellison-Loschmann L, Pattemore PK, Asher MI, et al. Ethnic differences in time trends in asthma prevalence in New Zealand: ISAAC Phases I and III. *Int J Tuberc Lung Dis* (2009) **13**: 775-82.
3. McCallum GB, Morris PS, Wilson CC, et al. Severity scoring systems: Are they internally valid, reliable and predictive of oxygen use in children with acute bronchiolitis? *Pediatr Pulmonol* (2012) **48**: 797-803.
4. Lambert SB, Ware RS, Cook AL, et al. Observational Research in Childhood Infectious Diseases (ORChID): a dynamic birth cohort study. *BMJ Open* (2012) **2**:e002134.

2.1 Supplementary Tables

Supplementary Table-1. Validated clinical severity score[3]

Points	Respiratory Rate (breaths/min)	Wheezing	SpO2 (in air after 1 min)	Accessory respiratory muscle utilisation
0	<30	None	>95	None
1	30-45	Expiration only	94-95	+
2	46-60	Entire expiration and inspiration with stethoscope only	90-93	++
3	> 60	Entire expiration and inspiration without stethoscope	<89	+++

Definitions

Points	Component	Instructions
	Respiratory rate (breaths per minute)	Count the number of time the infant's chest rises and falls for a period of 60-seconds
0<br>1<br>2<br>3	< 30<br>30-45<br>45-60<br>>60	
 0<br>1<br>2<br><br>3	Wheezing<br>None<br>Expiration only<br>Entire expiration and inspiration with stethoscope only<br>Entire expiration and inspiration without stethoscope	 Nil wheezing heard<br>Wheezing heard on expiration<br>Wheezing on inspiration and expiration<br><br>Wheezing on inspiration and expiration observed (standing at bedside)
 0<br>1<br>2<br>3	SpO_2<br>>95<br>94-95<br>90-93<br><89	Oxygen saturation in blood measured by pulse oximeter
 0<br>1<br>2<br>3	Accessory respiratory muscle utilisation<br>None<br>+<br>++<br>+++	Work of breathing assessed when off oxygen<br>No chest in-drawing i.e. absence of lower chest wall (intercostal) retraction during inhalation<br>(mild) = presence of mild intercostal retraction (just visible), no head bobbing or tracheal tug<br>(moderate) = moderate amount of intercostal retraction, no head bobbing or tracheal tug<br>(severe) = moderate or marked intercostal retraction with presence of head bobbing or tracheal tug

WHO definition of chest retraction = chest in drawing in a calm child, lower part of the chest moves in or retracts when inhalation occurs (i.e. all or none phenomena)

Supplementary Table-2. Subgroup analysis of hospital length of stay (LOS) until 'ready for hospital discharge'

	Median LOS in hours (IQR)			
	Azithromycin n=106	Placebo n=113	Difference (Azithromycin-placebo) (95% CI)	Interaction P-value
**Age**				
**≤ 6-months**	56 (40, 82)	64 (44, 96)	8 (-4, 21)	0.1
**> 6-months**	53 (46, 70)	52 (40, 63)	-1 (-11, 4)	
**Oxygen supplement requirement**				
**Yes**	56 (40, 78)	54 (40, 92)	-2 (-9, 11)	0.8
**No**	53 (46, 73)	56 (44, 78)	3 (-6, 11)	
**Location**				
**Remote**	55 (46, 76)	62 (44, 91)	7 (-13, 14)	0.8
**Urban**	53 (38, 62)	50 (38, 69)	-3 (-10, 11)	
**Antibiotics in hospital**				
**Study drug + beta-lactam**	54 (44, 82)	62 (45, 95)	8 (-4, 17)	0.06
**Study drug only**	53 (41, 62)	48 (38, 64)	-5 (-9, 8)	
**Respiratory hospitalisation prior to enrolment**				
**Yes**	54 (49, 75)	54 (37, 74)	0 (-21, 9)	0.4
**No**	53 (40, 74)	54 (43, 82)	1 (-5, 11)	

Supplementary Table-3. Subgroup analysis of time of oxygen requirement (where applicable)

	Median duration of oxygen in hours (IQR)			
	Azithromycin n=59	Placebo n=74	Difference (Azithromycin-placebo) (95% CI)	Interaction P-value
**Age**				
**≤ 6-months**	37 (24, 73)	50 (24, 80)	13 (-9, 23)	0.1
**> 6-months**	41 (30, 59)	28 (21, 39)	-13 (-21, -1)	
**Location**				
**Remote**	41 (24, 77)	35 (22, 76)	-6 (-16, 11)	0.6
**Urban**	38 (20, 52)	35 (26, 53)	-3 (-7, 12)	
**Antibiotics in hospital**				
**Study drug + beta-lactam**	41 (26, 77)	42 (26, 77)	1 (-13, 16)	0.3
**Study drug only**	38 (24, 50)	29 (20, 44)	-9 (-16, 7)	
**Respiratory hospitalisation prior to enrolment**				
**Yes**	54 (35, 77)	26 (16, 33)	-28 (-46, -2)	0.1
**No**	37 (23, 62)	38 (24, 72)	1 (-9, 12)	

Supplementary Table-4. Subgroup analysis of respiratory-related rehospitalisation within 6-months of discharge

	Azithromycin n/N (%)	Placebo n/N (%)	Odds Ratio (95%CI)	Interaction P-value
**Age**				
**≤ 6 months**	12/55 (22%)	14/61 (23%)	0.9 (0.4, 2)	0.2
**> 6 months**	19/50 (38%)	11/52 (21%)	2.3 (1.0, 5)	
**Oxygen supplement requirement**				
**Yes**	20/56 (36%)	15/66 (23%)	2 (1, 4)	0.4
**No**	11/49 (22%)	10/47 (21%)	1 (0.4, 3)	
**Location**				
**Remote**	21/74 (28%)	15/71 (21%)	2 (0.7, 3)	0.9
**Urban**	10/31 (32%)	10/42 (24%)	2 (0.6, 4)	
**Antibiotics in hospital**				
**Study drug + beta-lactam**	19/64 (30%)	13/68 (19%)	2 (0.8, 4)	0.5
**Study drug only**	12/41 (29%)	12/45 (27%)	1.1 (0.4, 3)	
**Respiratory hospitalisation prior to enrolment**				
**Yes**	11/18 (61%)	5/20 (25%)	5 (1, 18)	0.07
**No**	20/87 (23%)	20/93 (22%)	1 (0.5, 2)	

Supplementary Table-5.

	Azithromycin vs placebo	P-value
	**Multiplicative effect on geometric mean* (95% CI)**	
**Length of stay (hours) (unadjusted)**	0.9 (0.8, 1.1)	0.4
**Length of stay (hours) (adjusted)**	0.9 (0.8, 1.1)	0.2
**Time receiving oxygen (hours) (unadjusted)**	0.9 (0.7, 1.3)	0.9
**Time receiving oxygen (hours) (adjusted)**	1.0 (0.7, 1.3)	0.8
	Odds Ratio (95%CI)	P-value
**Respiratory rehospitalisation (unadjusted)**	1.5 (0.8, 3.0)	0.2
**Respiratory rehospitalisation (adjusted)**	1.2 (0.6, 2.3)	0.5

One person was missing household smoke status and was excluded from the above analyses

2.2 Supplementary Figures

Supplementary Figure-1. Frequency and distribution of viruses and atypical bacteria detected in nasopharyngeal swabs: Baseline and 48-hours later

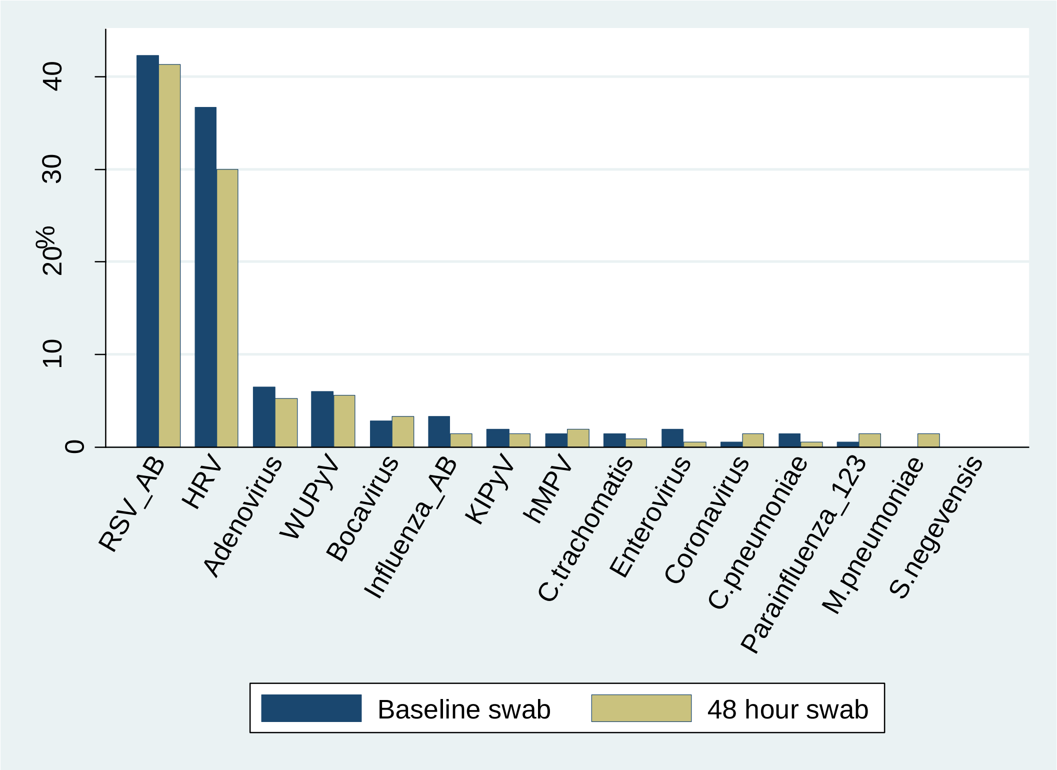

Supplement: Supplementary file 2 [file Data_Sheet_1.PDF]
